# Abdominal Stent Graft Numerical Models to Virtually Simulate Endovascular Aortic Repair: A Scoping Review

**DOI:** 10.1016/j.ejvsvf.2026.02.001

**Published:** 2026-02-12

**Authors:** Giulia de Campo, Jasper F. de Kort, Nesar A. Hasami, Anna Ramella, Sara Barati, Giulia Luraghi, Joost A. van Herwaarden, Francesco Migliavacca, Santi Trimarchi

**Affiliations:** a Department of Chemistry, Materials and Chemical Engineering “G. Natta”, Politecnico di Milano, Milan, Italy; b Section of Vascular Surgery, Cardio Thoracic Vascular Department, Fondazione IRCCS Ca’ Granda Ospedale Maggiore Policlinico, Milan, Italy; c Department of Vascular Surgery, University Medical Centre Utrecht, Utrecht, the Netherlands; d Department of Cardiothoracic Surgery, Radboud University Medical Centre, Nijmegen, the Netherlands; e Department of Clinical Sciences and Community Health, University of Milan, Milan, Italy

**Keywords:** Abdominal aortic aneurysm, Computational fluid dynamics (CFD), Endovascular aortic repair (EVAR), Finite element analysis (FEA), Patient specific simulation

## Abstract

**Introduction:**

Endovascular aortic repair (EVAR) is a common procedure used to treat abdominal aortic aneurysms. This scoping review aimed to identify the numerical methods currently available in the literature to analyse the surgical applicability and credibility of this procedure from a structural and fluid dynamic perspective.

**Method:**

PubMed (MEDLINE), Scopus, and Web of Science were systematically searched. The Preferred Reporting Items for Systematic Reviews and Meta-Analyses Extension for Scoping Reviews were followed. Data were analysed and summarised in tables. Quality assessment was performed using a 14 item rating rubric.

**Results:**

This scoping review identified 28 articles published in the last 10 years. Most of these articles were of intermediate to good quality. Seventeen articles (61%) focused on post-EVAR haemodynamics, performing computational fluid dynamics simulations. Six articles (21%) aimed to develop new methods to reproduce the EVAR procedure through finite element analysis. Five articles (18%) addressed the coupling between fluids and structures by performing a fluid–structure interaction simulation of the EVAR procedure. Studies using computational fluid dynamics demonstrated a more homogeneous approach in the selection of blood models, discretisation, and boundary conditions. Studies using finite element analysis adopted different strategies to reproduce the EVAR procedure *in silico*, with no common methodology identified. The choice of material properties assigned to the aorta, graft, and stent was highly heterogeneous. Four studies (16%) on fluid–structure interaction published in the last 10 years were identified. A Newtonian model for the blood was adopted in all the articles (100%), and in three articles (75%), a velocity waveform was applied as inlet boundary condition. For the structural part, more heterogeneities were found.

**Conclusion:**

This review provides an overview of the existing computational models to reproduce the EVAR procedure. Although promising, these simulation techniques remain largely in the developmental phase, and robust clinical validation is lacking. Future studies should incorporate clinically relevant outcome data to enhance the credibility and translational value of these models.

## Highlights

• CFD dominates EVAR simulations, showing consistency in methods and input parameters.
• FEA and FSI studies lack standardisation in geometry, materials, and setup.
• Most models use patient specific post-EVAR CT scans but lack clinical validation.
• Digital twins may aid stent graft sizing and predict EVAR complications.
• *In silico* EVAR tools show promise for patient specific surgical pre-planning.

## INTRODUCTION

Endovascular aortic repair (EVAR) is a common, minimally invasive procedure used to treat abdominal aortic aneurysms by deploying a stent graft to isolate the aneurysm from blood flow.^1^ Variability in pre-operative measurements, arising from inter- and intra-operator differences, can result in inconsistent sizing and positioning of the stent graft.^2^ These variations may affect apposition, oversizing, and the mechanical interaction between the device and the aortic wall. This can lead to post-operative complications, such as endoleak, stent graft migration, subsequent development of aortic dilatation graft kinking, or stent graft fracture.^3^ This is why, in recent years, computational modelling has been developed as a potentially valuable tool for clinicians in the pre-operative decision making phase.^3^

These *in silico* studies can be divided into computational fluid dynamics (CFD), structural analysis (finite element analysis (FEA), and fluid–structure interaction (FSI) analysis (Supplementary Table S1). CFD simulates the haemodynamics of the aorta, with both vessel wall and stent graft considered as rigid structures. FEA models the structural mechanics of the aorta and the stent graft, and FSI captures the interaction between fluid and structure, allowing analysis of aortic deformation under blood pressure.^4^

The objective was to identify current gaps and opportunities for improvement in future *in silico* studies, ultimately supporting the development of clinically reliable tools for patient specific planning of the EVAR procedure, such as stent graft selection and deployment, while also offering the potential to predict post-operative complications and improve long term outcomes.

## STUDY DESIGN

This analysis was conducted in alignment with the guidelines outlined in the Preferred Reporting Items for Systematic Reviews and Meta-Analyses Extension for Scoping Reviews (PRISMA-ScR), as well as established methodological frameworks for scoping reviews.^5^ In accordance with PRISMA-ScR, critical appraisal of the individual sources of evidence was considered optional.

## METHODS

### Objectives

The primary aim of this study was to compare different numerical models to reproduce the EVAR procedure *in silico*. The studies were analysed based on their similarities and differences in baseline characteristics, methodological approaches, numerical methods used, and outcomes.

### Literature sources and search strategy

The search process was conducted independently by two authors (G.d.C. and J.F.d.K.) with expertise in bioengineering and medicine. This process involved performing the systematic search, selection of studies based on inclusion and exclusion criteria, data extraction, and management of the collected information. In the event of discrepancies, a third and or fourth senior author (F.M. and S.T.) was consulted to reach a consensus. The systematic search was carried out on 31 July 2024 across three major databases: PubMed (MEDLINE), Scopus, and Web of Science. An updated search was performed on the 15 October 2025. No filters were applied beyond limiting the results to articles from the last ten years and in the English language. The search was structured around two main categories: computational simulation (e.g., virtual, simulation∗, FEA, and computational simulation) and EVAR (e.g., EVAR, abdominal stent graft, and aortic endograft). These categories included multiple entry terms and Medical Subject Headings terms, ensuring the complete capture of relevant studies. Within categories, terms were combined using the OR operator, while the two main categories were linked with AND. The final PubMed search string was then adapted for Scopus and Web of Science to maintain consistency across databases (Supplementary Table S2).

### Study selection

To streamline the selection process, Rayyan software ((Rayyan Systems Inc., Cambridge, MA, USA; web version, accessed July 2024) was used.^6^ No artificial intelligence functionalities were applied, and no automation tools were employed throughout this process. Furthermore, the reference lists of the included studies were examined to uncover any additional relevant studies that may not have been identified in the initial search. The study selection phase was completed on 30 September 2024. An updated search was performed on 15 October 2025.

### Inclusion and exclusion criteria

Original English language articles presenting a structural, fluid dynamic or FSI simulation of the EVAR procedure were selected. Exclusion criteria were as follows: (1) review article; (2) referral to the same methodology of another study that initially developed the EVAR simulation model; (3) fenestrated, branched, or otherwise complex EVARs; (4) models not simulating the stent graft, but only the aorta and or aneurysm; (5) models not simulating the aorta, but only the stent graft; (6) models only simulating the guidewire or catheter; (7) studies presenting real life simulations for clinical training purposes (i.e., non-numerical simulations).

### Data acquisition

Relevant data from the included studies were extracted by the authors (G.d.C. and J.K.) and organised into pre-defined tables using Microsoft Excel (Microsoft Corp, Redmond, WA) to group the data, enabling easy comparison across studies. Information was gathered on study characteristics (first author, publication year, article type, study location, and study aim), methodological details including clinical data where applicable (e.g., patient demographics, type of aortic disease, stent graft dimensions, and oversizing), or numerical data (e.g., segmentation input, computational methods, aortic and stent graft models, and other simulation components). Additionally, qualitative and quantitative clinical, numerical, or comparative outcomes were noted. Qualitative and quantitative outcomes were categorised into haemodynamic parameters (e.g., flow distribution and wall shear stress), structural parameters (e.g., displacement forces and seal zone stresses), and clinical endpoints (e.g., endoleak, migration, and thrombosis) to allow consistent comparison across studies.

### Quality assessment

The quality assessment was carried out independently by two authors (G.d.C. and J.F.d.K.), who evaluated each study using a 14 item rating rubric.^7^ To ensure consistency and accuracy, any discrepancies between the reviewers were resolved by a senior author (F.M.). Given the lack of a standardised quality assessment tool for computational modelling in vascular interventions, an adapted 14 item version of the existing simulation research rubric was used to enable a structured and quantitative evaluation of study quality. The studies were then categorised by quality: a score <50% indicated low quality, a score between 50% and 70% indicated intermediate quality, and a score >70% indicated high quality.

### Data presentation

Data are presented in text format, with numerical values reported as number (*n*) and percentage (%). Any missing data are indicated using a dash (−).

## RESULTS

### Study selection

The initial database search across three major databases yielded 1 153 articles. Following the removal of duplicates, 735 unique articles remained for title and abstract screening, which led to the exclusion of 611 articles that did not meet the pre-defined eligibility criteria, leaving 121 articles for full text review.

**Figure 1:**
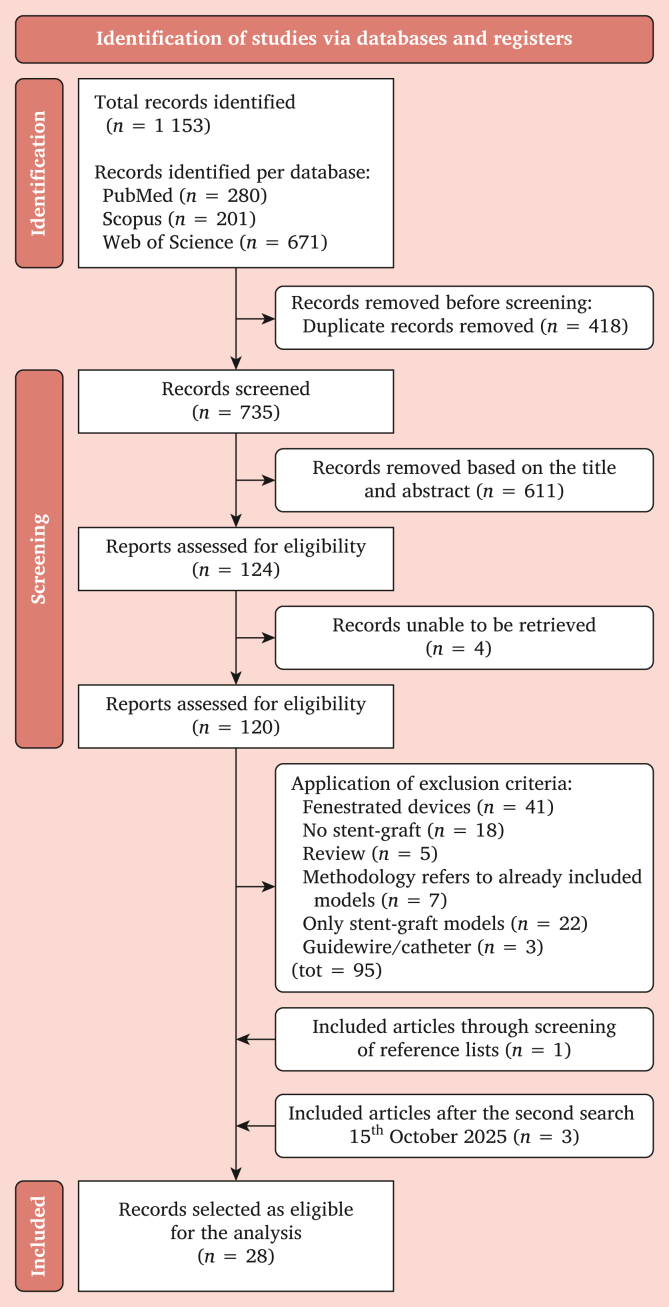
Flowchart of inclusion and exclusion criteria.

Following full text screening, 26 articles met the inclusion criteria. An additional study was identified through reference list screening, bringing the total to 27.^8^, ^9^, ^10^, ^11^, ^12^, ^13^, ^14^, ^15^, ^16^, ^17^, ^18^, ^19^, ^20^, ^21^, ^22^, ^23^, ^24^, ^25^, ^26^, ^27^, ^28^, ^29^, ^30^, ^31^, ^32^, ^33^, ^34^ However, as the study by Kyriakou *et al.*^13^ included both FEA and CFD analyses, it was counted as two separate entries, resulting in a final total of 28 studies. The full PRISMA-ScR flow diagram is shown in Fig. 1.

### Study characteristics

Table 1 provides a detailed description of the studies included in this review. Notably, 21 studies (75% [21 of 28])^8^^,^^10^, ^11^, ^12^, ^13^, ^14^, ^15^, ^16^^,^^18^^,^^20^^,^^21^^,^^23^^,^^24^^,^^27^^,^^28^^,^^30^, ^31^, ^32^, ^33^, ^34^ were published in engineering focused journals, while four articles (14% [4 of 28])^9^^,^^12^^,^^25^^,^^26^ were published in clinically focused journals.

**Table 1:** Study characteristics of the 28 articles analysed, including computational fluid dynamics (CFD), finite element analysis (FEA), and fluid–structure interaction (FSI) simulations of the endovascular aortic repair (EVAR) procedure, aim of the study, and quality assessment.

Author, year	Place	Journal focus	Simulation type	Aim of the study	Software used	Parameter evaluated	Quality assessment score
Georgakarakos *et al.*,^19^ 2014	Greece	Engineering	CFD	Evaluation and comparison of flow dynamics, pressure distribution and shear forces between cross limb design and traditional bifurcated configuration.	ANSYS Fluent (Ansys, Inc., Canonsburg, PA, USA)	Velocity, pressure, and WSS	38 (low)
Stefanov *et al.*,^15^ 2016	Ireland	Engineering	CFD	Evaluation of the haemodynamic behaviour of blood flow in crossed and non-crossed SG limbs and in pre-operative AAA.	ANSYS Fluent (Ansys, Inc., Canonsburg, PA, USA)	Maximum velocity, maximum deceleration, drag force, Dean number	68 (intermediate)
Polanczyk *et al.*,^29^ 2016	Poland	Multidisciplinary	CFD	Estimation of the impact of the SG on WSS using CFD simulations.	ANSYS Fluent (Ansys, Inc., Canonsburg, PA, USA)	WSS, shape factors	69 (intermediate)
Aristokleous *et al.*,^30^ 2016	Greece	Engineering	CFD	Evaluation of the influence of the stenosis caused by the sealing of the O-ring of the Ovation SG.	ANSYS Fluent (Ansys, Inc., Canonsburg, PA, USA)	WSS, wall shear rate, nominal values of the radii, average pressure, peak systolic pressure, velocity	75 (high)
Looyenga *et al.*,^31^ 2017	USA	Engineering	CFD	Evaluation of the effect of stent struts on blood flow patterns that can cause blood platelet adhesion and blood clotting.	Simcenter STAR-CCM+ (Siemens Digital Industries Software, Plano, TX, USA)	WSS, velocity, OSI, TAWSS, RRT, and WSS average on the cardiac cycle	50 (intermediate)
Raptis *et al.*,^21^ 2017	Greece	Engineering	CFD	Definition of the haemodynamics in Endurant and Excluder SGs.	ANSYS Fluent (Ansys, Inc., Canonsburg, PA, USA)	Maximum WSS, velocity at peak systole, mean helicity at mid diastole, DFs	54 (intermediate)
Raptis *et al.*,^17^ 2018 Fare clic o toccare qui per immettere il testo.	Greece	Multidisciplinary	CFD	Assessment of the haemodynamics present in the AFX and Nellix endografts to give an outline of the strength and the weak haemodynamic features of both SGs.	ANSYS Fluent (Ansys, Inc., Canonsburg, PA, USA)	Pressure drop, velocity, helicity, WSS	56 (intermediate)
Liu *et al.*,^20^ 2018	China	Engineering	CFD	Evaluation of the haemodynamics on cross limb SG changing the cross angle and the cross position in order to find the optimal configuration, using CFD simulations.	ANSYS Fluent (Ansys, Inc., Canonsburg, PA, USA)	Helicity, TAWSS, OSI, RRT, flow pattern	69 (intermediate)
Kyriakou *et al.*,^13^ 2020	UK	Engineering	CFD	Performing a CFD analysis starting from the output of the FEA analysis of the Anaconda SG placement.	Abaqus (Dassault Systèmes SIMULIA Corp., Johnston, RI, USA)	Velocity field, vorticity, TAWSS, OSI, RRT	85 (high)
Domanin *et al.*,^25^ 2020	Italy	Clinical	CFD	Comparison of 4D dynamic CTA with a CFD analysis to see if it can predict the relationship between graft movement and viscous blood forces.	LifeV (open-source; available at lifev.org)	WSS, TAWSS, FUD (FUD between first and fifth year of follow up) and IHD (endograft IHD)	89 (high)
Qing *et al.*,^22^ 2021 Fare clic o toccare qui per immettere il testo.	China	Physiology	CFD	Investigation of the influence of the cross limb SG on both near wall and intragraft flow dynamics, evaluating the impact of neck angulation on local flow feature using CFD analysis.	ANSYS Fluent (Ansys, Inc., Canonsburg, PA, USA)	Flow field, pressure drop, WSS, helicity	65 (intermediate)
Ashraf *et al.*,^26^ 2021	South Korea	Clinical	CFD	Analysis of the haemodynamic behaviour of crossed and non-crossed limb SG implanted with the top down approach and a non-crossed limb SG implanted with the bottom up technique.	COMSOL Multiphysics (COMSOL, Inc., Burlington, MA, USA)	TAWSS, WSS, OSI, fluid streamlines, helicity	69 (intermediate)
Teng *et al.*,^24^ 2023	China	Engineering	CFD	Development of an uniform model for different sexes to calculate the DF acting on the SG using CFD. The goal was to explore the differences in the vasculature of male and female AAA and how it influences the DFs.	ANSYS Fluent (Ansys, Inc., Canonsburg, PA, USA)	DF, area average DF, velocity, pressure, and WSS	48 (low)
Qing *et al.*,^23^ 2022	China	Engineering	CFD	Comparison of the DFs measured on patient specific anatomies by performing CFD analysis and using a simpler approach proposed in the article.	ANSYS Fluent (Ansys, Inc., Canonsburg, PA, USA)	DF	65 (intermediate)
Polanczyk *et al.*,^27^ 2022	Poland	Engineering	CFD	Relate the influence of the SG spatial configuration on endoleak formation, under realistic blood flow.	ANSYS Fluent (Ansys, Inc., Canonsburg, PA, USA)	Blood velocity profile, blood pressure, and WSS	84 (high)
Brand *et al.*,^28^ 2023	Israel	Engineering	CFD	Assessment of the relationship between SG curvature and the DFs in patient specific models.	ANSYS Fluent (Ansys, Inc., Canonsburg, PA, USA)	DF, SG curvature based on the local radius (CLC-R) or based on the similarity to a straight branch, area ratio between the inlet and the outlet of the bifurcation region, length and surface area of the graft.	56 (intermediate)
Zhang *et al.*,^34^ 2024	China	Engineering	CFD	Evaluation of risk of intraluminal prosthetic graft thrombus formation associated with the aortic morphology	ANSYS Fluent (Ansys, Inc., Canonsburg, PA, USA)	Flow velocity, OSI, WSS, TAWSS, RRT	48 (low)
Perrin *et al.*^18^ 2015	France	Engineering	FEA	Development of a new methodology to reproduce the EVAR procedure through FEA simulations.	Abaqus (Dassault Systèmes SIMULIA Corp., Johnston, RI, USA)	Qualitative and quantitative comparison between simulation result and CT–post	75 (high)
Hemmler *et al.*,^14^ 2018	Germany	Engineering	FEA	Investigation of SG oversizing, the SG pre-deformation and its impact on SG stress and contact with the aorta.	In house	SG pre-deformation, SG oversizing	82 (high)
Pocivavsek and Milner,^12^ 2020	USA	Clinical	FEA	Evaluation of the AAA repair after EVAR, analysing both the pre-operative anatomy and endograft stability post-surgery.	Abaqus (Dassault Systèmes SIMULIA Corp., Johnston, RI, USA)	Model validation and evaluation of the cohesive forces to understand the SG sealing	67 (intermediate)
Kyriakou *et al.*,^13^ 2020	UK	Engineering	FEA	Anaconda device placement and deployment inside the aorta using an FEA simulation.	Abaqus (Dassault Systèmes SIMULIA Corp., Johnston, RI, USA)	Comparison of the sections between simulation and experiment in the distal and proximal section.	75 (high)
Pionteck *et al.*,^16^ 2020	France	Engineering	FEA	Proposal of a method that can be fast and accurate to provide a proper prediction of the SG positioning.	In house	Comparison between simulations and CT post-procedure	77 (high)
Abdollahi *et al.*,^32^ 2025	Canada	Engineering	FEA	Presentation of a new workflow to simulate EVAR implantation, with particular attention on the introduction of the catheter with the SG inside the vessel.	LS-DYNA (Ansys, Inc., Canonsburg, PA, USA)	Radius differences between pre-operative and post-operative CT after EVAR implantation	85 (high)
Lu *et al.*,^11^ 2016	Taiwan	Engineering	FSI	Prediction of the position of endoleak formation in post-operative EVAR patients using FSI.	ADINA (Bentley Systems, Inc., Exton, PA, USA)	Von Mises stress distribution to analyse the impact of flow on the SG. Correlation between endoleak and peak von Mises stress.	46 (intermediate)
Jayendiran *et al.*,^9^ 2020	Qatar	Clinical	FSI	Estimation of the effect of flow on the mechanical behaviour of the aorta and SG, performing an FSI simulation on a honeycomb SG.	Abaqus (Dassault Systèmes SIMULIA Corp., Johnston, RI, USA)	Strain during the crimping and release phase of the SG. Von Mises stress on the SG after expansion using an FSI simulation	67 (intermediate)
Bologna *et al.*,^8^ 2023	Italy	Engineering	FSI	Development of a patient specific FSI simulation to evaluate flow pattern, pressure and WSS to understand the benefit of using patient specific SG	-	CFD: WSS, pressure, velocity, and the flow pattern at peak systole FEA: Wall stress distributions FSI: pressure, wall stress	75 (high)
Xie *et al.*,^10^ 2024	China	Engineering	FSI	Evaluation of the haemodynamic performance of different cross limb features using a one way FSI analysis.	COMSOL Multiphysics (COMSOL, Inc., Burlington, MA, USA)	Flow pattern, helicity strength, DF, and WSS	55 (intermediate)
Mo *et al.*,^33^ 2025	China	Engineering	FSI	Evaluation of type II endoleak in an idealised vessel using FSI and CFD simulations.	-	Flow velocity, volume flow rate, aneurysm sac pressure, WSS	56 (intermediate)

AAA = abdominal aortic aneurysm; CFD = computational fluid dynamics; CLC = centreline curvature; CT = computed tomography; CTA = computed tomography angiography; DF = displacement force; EVAR = endovascular aortic repair; FEA = finite element analysis; FSI = fluid–structure interaction; FUD = follow up displacement; IHD = intra heartbeat displacement; OSI = oscillatory shear index; RRT = relative residence time; SG = stent graft; TAWSS = time average wall shear stress; WSS = wall shear stress.

**Table 2:** Methodological aspect of the computational fluid dynamics (CFD) simulations, focusing on the model discretisation, boundary conditions, and conclusions of the articles.

Author	Input data	Model discretisation	BCs and fluid modelling	Parameter analysed	Conclusion
Georgakarakos *et al.*,^19^ 2014	One patient specific CT and one idealised model	-	Blood: non-Newtonian (Carreau-Yasuda model) Inlet BC: velocity waveform Outlet BC: pressure waveform	Velocity, pressure, and WSS	After EVAR, lower systolic and diastolic pressure peaks were observed, due to a wall stiffness increase. The cross limb design seemed to reduce aortic neck acuity and iliac limb angulation. Velocity streamlines showed flow disturbances at the inlet, with the cross limb configuration being more prone to vortex formation. Shear stress values were similar between the models.
Aristokleous *et al.*,^30^ 2016	Four patient specific post-operative CTs	Hexahedral element	Blood: Newtonian Inlet BC: physiological flow waveform Outlet BC: flow split waveform	WSS, wall shear rate, nominal values of the radii, average pressure, peak systolic pressure, velocity	Ovation endograft was used to treat challenging anatomies. The flow reached up to 8 fold, and vortex formation was present in high WSR regions. This may increase the probability of thrombus formation.
Polanczyk *et al.*,^29^ 2016	27 patient specific CTs post-EVAR	Tetrahedral elements	Blood: non-Newtonian (Quemada model) Inlet BC: velocity waveform (from USG doppler) Outlet BC: constant pressure	WSS, shape factors	More twisted SGs lead to higher forces and an increased risk of implant migration. Iliac artery angulations can reduce flow and create turbulence. Deformation of the SG bifurcation can damage blood flow, increasing WSS values. Tortuosity in the SG can increase the risk of implant failure due to the rising pushing forces.
Stefanov *et al.*,^15^ 2016	12 patient specific anatomies	Multi-block O-grid mesh	Blood: non-Newtonian (Carreau-Yasuda model) Inlet BC: physiological flow rate waveform (from USG doppler) Outlet BC: physiological pressure waveform	Maximum velocity, maximum deceleration, drag force, Dean number	Dean number can be used to predict the blood flow pattern. Crossed SG devices promote favourable haemodynamics by decreasing recirculation region. In crossed SG, the acting drag force is decreased due to a spiral flow.
Looyenga *et al.*,^31^ 2017	Two idealised models	Prism, polyhedral, and surface elements	Blood: Newtonian Inlet BC: pulsatile flow waveform Outlet BC: flow split waveform	WSS, velocity, OSI, TAWSS, RRT, and WSS average on the cardiac cycle	The stent struts caused flow disruption. Recirculation was observed determined by an OSI >0.3. The WSS results indicated the possibility of facture area formation on the struts. RRT pointed out small movements that can lead to plaque adhesion.
Raptis *et al.*,^21^ 2017	20 patient specific CT–post	Tetrahedral elements	Blood: Newtonian Inlet BC: physiological pressure waveform Outlet BC: physiological flow rate waveform	Maximum WSS, velocity at peak systole, mean helicity at mid diastole, displacement forces	Excluder generated significantly higher velocity, WSS, and stronger displacement forces than Endurant. Both devices seemed to promote the development of secondary flows. Stronger DF but a more efficient flow was found in Excluder compared with Endurant, which instead had higher secondary flows.
Liu *et al.*,^20^ 2018	Ten idealised models	Surfaces were meshed using a mixture of tetrahedral and hexahedral elements	Blood: non-Newtonian (Carreau-Yasuda model) Steady simulation: Inlet BC: constant pressure Outlet BC: constant velocity Pulsatile simulation: Inlet BC: velocity waveform Outlet BC: pressure waveform	Helicity, TAWSS, OSI, RRT, flow pattern	Increasing the cross angle and decreasing the cross position ratios, the intensity of the helical flow strength was reduced. As the cross angle increased, the TAWSS remained the same, while OSI and RRT decreased. Lower cross position and larger cross angles correlate with stronger displacement forces acting on the SG, thus increasing the migration likelihood.
Raptis *et al.*,^17^ 2018	16 patient specific CT–post	Tetrahedral elements	Blood: Newtonian Inlet BC: pulsatile pressure waveform Outlet BC: physiological flow rate waveform	Pressure drop, velocity, helicity, WSS	The AFX endograft with infrarenal fixation appears to restore blood flow. However, secondary flows appeared in the upper section of the graft and a pressure drop in its limbs. Similarly, the Nellix graft also presented haemodynamic efficiency.
Domanin *et al.*,^25^ 2020	Two patient specific CT–post	Tetrahedral elements	Blood: Newtonian Inlet BC: physiological flow rate waveform Outlet BC: constant pressure	WSS, TAWSS, FUD (FUD between first and fifth year of follow up) and IHD (endograft intra heartbeat displacement)	A significant permanent deformation over the years, evaluated by FUD, was observed for the endograft analysed. High IHD corresponded to regions with high WSS and vice versa for lower values of IHD. TAWSS was high in the region of maximum FUD. Systolic WSS was higher whereas IHD was not negligible. Low viscous forces corresponded to remodelling regions. High values of FUD were associated with blood vortex generation.
Kyriakou *et al.*,^13^ 2020	One idealised model of an abdominal aneurysm	Tetrahedral elements	Blood: non-Newtonian (Carreau-Yasuda model) Inlet BC: parabolic velocity profile Outlet BC: pressure waveform	Velocity field, vorticity, TAWSS, OSI, RRT	Including graft wrinkles in the model resulted in a lower WSS thus indicating the possibility of thrombus formation.
Ashraf *et al.*,^26^ 2021	Three idealised geometries taken from three patient specific CT–post	-	Blood: non-Newtonian (Carreau-Yasuda model) Inlet BC: physiological velocity waveform Outlet BC: pressure waveform	TAWSS, WSS, OSI, fluid streamlines, helicity	In the crossed SG, OSI and helicity were higher than in the non-crossed approach, which led to a reduction of cell adhesion. Displacement forces were much higher in the crossed SG than in the other two non-crossed SGs. This might cause fatigue failure over time.
Qing *et al.*,^22^ 2021	26 idealised models	Combination of unstructured tetrahedral elements and structured hexahedral elements	Blood: non-Newtonian (Carreau-Yasuda model) Inlet BC: pulsatile velocity waveform Outlet BC: Windkessel model	Flow field, pressure drop, WSS, helicity	As the neck angle increased, the pressure dropped and the OSI rose while the TAWSS decreased for both the crossed and non-crossed configurations. Helicity is increased only in the crossed limb configuration with a coronal angulated neck. Cross limb configuration seemed not to increase thrombosis formation.
Polanczyk *et al.*,^27^ 2022	One patient specific anatomy type II endoleak and one idealised model without endoleak	Tetrahedral elements	Blood: non-Newtonian (Quemada model) Inlet BC: physiological flow waveforms Outlet BC: constant pressure	Blood velocity profile, blood pressure, and WSS	High blood velocity in an endoleak anatomy could lead to its gradual rupture. The presence of an endoleak lowers average blood velocity in the SG, potentially contributing to clot formation. However, no clot formation at the leak site was observed due to high WSS, which prevents thrombosis.
Teng *et al.*,^24^ 2023	One idealised model for men and one idealised model for women	Tetrahedral elements	Blood: Newtonian Inlet BC: velocity waveform Outlet BC: pressure waveform	Drug forces (DF), area average DF, velocity, pressure and WSS	DF was dependent on both vessel size and the curvature of the abdominal aorta. DF values were higher in male models, but the area averaged DF was greater in females, due to larger SGs. The stronger impact of pulsatile blood flow on female SGs may explain their higher complication rates after EVAR.
Brand *et al.*,^28^ 2023	Four patient specific CT–post	Tetrahedral shell elements, converted in polyhedral cells in Fluent	Blood: Newtonian Inlet BC: peak systolic mass flow Outlet BC: peak pressure	DF, stent graft curvature based on the local radius (CLC-R) or straight branch (CLC-D), area ratio between the inlet and the outlet of the bifurcation region, length and surface area of the graft	The total DF was more sensitive to changes in curvature than to the average curvature. The average CLC-D variance yielded the best estimate of DF. Migration can be assessed with the method proposed in the article.
Qing *et al.*,^23^ 2022	20 patient specific models	Combination of tetrahedral and hexahedral elements	Blood: Newtonian Inlet BC: constant velocity Outlet BC: peak pressure	DF	Angulation of the proximal and distal SG significantly impacts the total DF. If the angulations differ greatly, the DF increases. Therefore, minimising the angle difference between the SG ends the risk of displacement after EVAR can be reduced.
Zhang *et al.*,^34^ 2024	Six idealised models	-	Blood: Non-Newtonian (Quemada model) Inlet BC: velocity waveform Outlet BC: pressure waveform	Flow velocity, WSS, OSI, RRT, TAWSS	Endograft compression and distortion were identified as potential risk factors for intraluminal prosthetic graft thrombus development.

BC = boundary condition; CLC, centreline curvature; CT = computed tomography; DF = displacement/drag force; EVAR = endovascular aortic repair; FUD = follow up displacement; IHD = intra heartbeat displacement; OSI = oscillatory shear index; RRT = relative residence time; SG = stent graft; TAWSS = time average wall shear stress; USG = ultrasonography; WSR = wall shear rate; WSS = wall shear stress.

In general, 17 articles (61% [17 of 28])^13^^,^^15^^,^^17^^,^^19^, ^20^, ^21^, ^22^, ^23^, ^24^, ^25^, ^26^, ^27^, ^28^, ^29^, ^30^, ^31^^,^^34^ were focused on post-EVAR haemodynamics, six (21% [6 of 28])^12^, ^13^, ^14^^,^^16^^,^^18^^,^^32^ on the development of finite element methods to reproduce the EVAR procedure, and five (18% [5 of 28])^8^, ^9^, ^10^, ^11^^,^^33^ concentrated on FSI simulations. Notably, only one study conducted an FEA followed by a CFD analysis.^13^

### Quality assessment

The quality assessment showed that three studies (11% [3 of 28])^19^^,^^24^^,^^34^ were of low quality, 14 studies (50% [14 of 28])^9^^,^^10^^,^^12^^,^^15^^,^^17^^,^^20^, ^21^, ^22^, ^23^^,^^26^^,^^28^^,^^29^^,^^31^^,^^33^ were of intermediate quality, and ten studies (36% [10 of 28])^8^^,^^13^^,^^14^^,^^16^^,^^18^^,^^25^^,^^27^^,^^30^^,^^32^ were of high quality (Table 1). Detailed scores for each study are provided in Supplementary Table S3.

### Clinical data

Twelve studies (43% [12 of 28])^8^^,^^11^^,^^12^^,^^15^^,^^16^^,^^18^^,^^19^^,^^21^^,^^23^^,^^29^^,^^30^^,^^32^ used patient specific geometry for their simulations; however, only six of these (21% [6 of 28])^11^^,^^16^^,^^18^^,^^29^^,^^30^^,^^32^ provided additional clinical data beyond just the number of patients. In these six studies, sex data were available for nine patients, all of whom were male. All patient specific geometry included infrarenal abdominal aortic aneurysms (Supplementary Table S4).

### CFD simulations

CFD articles were categorised by objectives into three main groups. Firstly, five studies (29% [5 of 17])^15^^,^^19^^,^^20^^,^^22^^,^^26^ investigated haemodynamics in the ‘ballerina’ configuration and its differences from the conventional procedure.^15^ (The ‘ballerina’ type or cross limb procedure consists of crossing the limb component of the EVAR device inside the pathological track of the aorta.) Secondly, two studies (12% [2 of 17])^17^^,^^21^ compared the haemodynamics of two commercial devices. The remaining ten studies (59% [10 of 17])^13^^,^^23^, ^24^, ^25^^,^^27^, ^28^, ^29^, ^30^, ^31^^,^^34^ aimed to evaluate how the stent graft influences various fluid dynamic parameters (see Table 2).

Although these studies had different goals, the choices regarding simulation settings were consistent. In nine studies (53% [9 of 17]),^15^^,^^17^^,^^19^^,^^21^^,^^23^^,^^25^^,^^27^^,^^28^^,^^30^ patient specific computed tomography (CT) scans were used, while nine studies (53% [9 of 17])^15^^,^^17^^,^^19^^,^^21^^,^^23^^,^^25^^,^^27^^,^^28^^,^^30^ employed idealised geometries. Only Kyriakou *et al.*^13^ used the endpoint of FEA simulation as the starting point of the CFD simulation, meaning that the device is not just the stent graft segmented from the post–CT scan, but a separate part compared with the aorta.

Blood was modelled as a Newtonian fluid in half of the studies^17^^,^^21^^,^^23^, ^24^, ^25^^,^^27^^,^^28^^,^^30^ and as non-Newtonian in the other half.^13^^,^^15^^,^^19^^,^^20^^,^^22^^,^^26^^,^^27^^,^^29^^,^^34^ Flow velocity was set as an inlet boundary condition in 15 of the studies (88% [15 of 17]),^13^^,^^15^^,^^19^^,^^21^, ^22^, ^23^, ^24^, ^25^, ^26^, ^27^, ^28^, ^29^, ^30^, ^31^^,^^34^ while in the remaining two (12% [2 of 17]),^17^^,^^20^ the pressure was set as inlet boundary. For the outlet, a pressure boundary condition was used in 12 studies (71% [12 of 17]),^13^^,^^15^^,^^19^^,^^20^^,^^23^, ^24^, ^25^, ^26^, ^27^, ^28^, ^29^^,^^34^ a flow split was used in two studies (12% [2 of 17]),^30^^,^^31^ a physiological flow rate was used in two other articles (12% [2 of 17]),^17^^,^^21^ and a Windkessel model was used in one study (6% [1 of 17]).^23^ Focusing on the CFD article results, in seven articles (41% [7 of 17]),^15^^,^^17^^,^^19^, ^20^, ^21^, ^22^^,^^26^ the authors compared the haemodynamics between crossed and non-crossed limb configurations or between different commercial devices.

The remaining CFD studies (59% [10 of 17])^13^^,^^23^, ^24^, ^25^^,^^27^, ^28^, ^29^, ^30^, ^31^^,^^34^ focused on various haemodynamic aspects of the flow within the aorta with an implanted stent graft. The specific results from these investigations are summarised in Table 2.

### FEA simulations

A detailed description of the stent graft model discretisation is reported in Supplementary Table S5. Stents were mostly discretised using beam elements (67% [4 of 6]),^13^^,^^16^^,^^18^^,^^32^ whereas grafts were modelled with shell elements (50% [3 of 6]).^13^^,^^18^^,^^32^ Regarding material modelling, for stainless steel rings, adopted in two articles (29% [2 of 7]),^14^^,^^16^ a linear elastic material was employed, while a shape memory material, used in one article (17% [1 of 6]),^18^ or a linear elastic material, used in three studies (50% [3 of 6]),^13^^,^^16^^,^^32^ was employed to model nitinol.

The graft fabric material was modelled with varying approaches across studies: as a linear orthotropic material (17% [1 of 6]),^18^ a compressible neo-Hookean model (17% [1 of 6]),^14^ an isotropic fabric model (17% [1 of 6]),^32^ and an elastic Hookean solid model (17% [1 of 6]).^12^

For aorta geometries, four studies (67% [4 of 6])^12^^,^^16^^,^^18^^,^^32^ used patient specific anatomies derived from CT scans, while the remaining two studies (33% [2 of 6])^13^^,^^14^ employed idealised geometries. For mesh type, shell elements were used in two studies (33% [2 of 6]).^18^^,^^32^ Other discretisations adopted were hexahedral or tetrahedral elements. Regarding material modelling, the aorta was treated heterogeneously by adopting a rigid material (17% [1 of 6])^13^ or a hyperelastic model (83% [5 of 6])^12^^,^^14^^,^^16^^,^^18^^,^^32^ (Supplementary Table S5).

In examining the EVAR procedure as a whole, five studies (83% [5 of 6])^12^, ^13^, ^14^^,^^18^^,^^32^ focused on developing the full EVAR process from device insertion to its expansion in the vessel by employing varied approaches. Two studies (33% [2 of 6])^14^^,^^18^ focused on the morphing procedure, whereas the other two studies (33% [2 of 6])^13^^,^^32^ aimed to reproduce the entire procedure including device pre-stress, crimping, tracking, and release. One study (17% [1 of 6])^16^ proposed a fast method for accurate EVAR outcome prediction, and another (17% [1 of 6])^12^ analysed the interaction between the aorta and the stent graft after deployment (Table 3).

**Table 3:** Methodological aspects of the finite element analysis (FEA) simulation focusing on the step followed to implement the simulation, the parameters evaluated and the conclusions.

Author	Aim of the study	Simulation steps	Parameter evaluated	Conclusion/clinical outcome
Perrin *et al.*^18^ 2015	Development of a new methodology to reproduce the EVAR procedure through FEA simulations.	Simulation steps: 0. Device prestress 1. Slight crimping 2. Insertion of the device in a virtual tubular shell 3. Displacement applied as boundary conditions to morph the geometry into the patient specific anatomy 4. Deployment through the release of the boundary conditions applied to the aorta	Qualitative and quantitative comparison between simulation result and CT–post	The method developed showed a good agreement with the CT–post, both qualitatively and quantitatively, for three patients.
Hemmler *et al.*,^14^ 2018	Investigation of the SG oversizing, the SG pre-deformation and its impact on SG stress and contact with the aorta.	The steps modelled were the following: 1. Vessel and SG pre-stress 2. SG placement: The placement of the device started with the alignment of the SG to the centreline of the vessel with a reduced SG diameter. 3. Deployment: Activating the contact between SG and the aorta, the device was released. A systolic pressure was applied to the vessel lumen and to the SG.	SG pre-deformation, SG oversizing	Tissue strain and stresses increased after device deployment in the proximal and distal apposition area due to the presence of thrombus or calcifications. SG expansion diameter is a good tool to analyse SG oversizing. The device pre-stress is stronger with a highly angulated aortic neck.
Kyriakou *et al.*,^13^ 2020	Anaconda device placement and deployment inside the aorta using an FEA simulation.	The simulation followed two main steps: 1. Device pre-stress: the connection between stent and graft is achieved through a sinusoidal displacement of the ring that then was attached to the graft using a frictionless contact with separation restriction. The ring was then pulled, and the contact was changed to rough to impose a secure fixation. 2. Deployment and release were reproduced using two catheters. The rings were compacted through a displacement into catheter A. The catheter moved on the centreline of the vessel. Once the device arrived at the target position, the contact switched to catheter B. Pressure was applied to catheter B to allow the device to expand inside the vessel.	Comparison of the sections between simulation and experiment in the distal and proximal section	The deployment model of the Anaconda SG showed good agreement with the CT–post in the proximal and distal regions of the endograft, while greater distances between simulation and CT–post were found in the aneurysm region, where the SG has no anatomic constraint.
Pionteck *et al.*,^16^ 2020	Proposal of a method that can be fast and accurate to provide a proper prediction of SG positioning.	The method consisted of: 1. A preliminary stage that identified the stent barycentre in the aorta and an axisymmetric reconstruction of the stent. To avoid complex boundary conditions, the SG model was pre-positioned in the aorta and then displacement was applied to the wrongly positioned SG to teach the locked position. (locked position = D_sg > D_aorta; free position if D_sg < D_aorta) At this stage, the SG was modelled as a succession of beam elements. 2. The second stage implied the rotation minimisation and the individual SG deployment. The stent was deployed in six min and using penalty based contact.	Comparison between simulations and CT–post	Development of a fast and accurate method to reproduce the EVAR procedure. This method can also model the fenestrated SG positioning.
Pocivavsek and Milner,^12^ 2020	Evaluation of the AAA repair after EVAR, analysing both the pre-operative anatomy and endograft stability post-surgery.	The aorta was fixed at the edges, and uniform pressure was applied to the internal lumen of the aorta. The aorta–stent interface carried stresses, which means that as the stress associated with the cohesive forces overcame the maximum cohesive stress, the two parts were not considered in contact anymore and failure occurred.	Model validation and evaluation of the cohesive forces to understand the SG sealing	The model described involved the balance between the aortic neck elastic energy and the adhesion energy of the seal zone. Stable distal sealing and unstable proximal sealing were found. This corresponded to the development in the patient of a type IA endoleak.
Abdollahi *et al.*,^32^ 2025	Development of a new methodology to reproduce the EVAR procedure through FEA simulations, with a particular attention on the delivery system.	The method consisted of: 0. Device pre-stress. 1. Device crimping in a catheter. 2. Device tracking by pushing the catheter in the vessel. The navigation is automatically defined by ‘sensors’ to activate or deactivate the prescribed insertion. 3. Complete release of the device in the region of interest. Both the main body and the limb component are reproduced.	Qualitative and quantitative comparison between simulation result and CT–post	The method applied reproduced all the steps of the clinical procedure with particular attention to the crimping procedure and the correct reproduction of the delivery system. A good comparison between simulation and post–CT of the patient was achieved.

AAA = abdominal aortic aneurysm; CT = computed tomography; EVAR = endovascular aortic repair; FEA = finite element analysis; SG = stent graft.

### FSI simulations

Supplementary Table S6 specifies FSI studies. Two studies (40% [2 of 5])^8^^,^^10^ used one way FSI simulation and three studies (60% [3 of 5])^9^^,^^11^^,^^33^ used two way FSI. One way FSI means that the fluid flow influences the aortic wall, but the aortic wall does not influence the fluid flow, whereas the two way FSI considers the mutual interaction between the blood and the aorta.^35^ Starting from the structural analysis, the stent graft was meshed with linear shell elements in two studies (40% [2 of 5])^9^^,^^11^ and with tetrahedral elements in two studies (40% [2 of 5]).^8^^,^^33^

Different material properties were considered across the studies; the graft was modelled as either anisotropic/isotropic hyperelastic^9^ or linear elastic material^11^ in one article each (20% [1 of 5]), while the stent was modelled as a shape memory material in two studies (40% [2 of 5]),^8^^,^^9^ as a hyperelastic model as the aorta in one study (20% [1 of 5]),^11^ and in the remaining two, the material was not specified (40% [2 of 5]).^10^^,^^33^

For the aorta, two studies (40% [2 of 5])^8^^,^^11^ adopted patient specific anatomies, two studies (40% [2 of 5])^9^^,^^33^ used an idealised model, and one study (20% [1 of 5])^10^ incorporated both. The aorta was discretised with shell or tetrahedral elements and modelled as hyperelastic in four studies (80% [4 of 5]),^8^^,^^9^^,^^11^^,^^33^ whereas in the remaining article (20% [1 of 5]),^8^ the material was not specified. Four studies (80% [4 of 5])^8^^,^^9^^,^^11^^,^^33^ represented the fluid as Newtonian and one as non-Newtonian (20% [1 of 5]).^33^ In three studies (60% [3 of 5]),^8^^,^^10^^,^^11^ a velocity waveform was applied at the inlet with a pressure condition at the outlet.

As with CFD studies, the FSI studies had varying objectives, which are summarised in Table 4, along with the boundary conditions adopted in each study.

**Table 4:** Methodology employed for the fluid–structure interaction (FSI) simulation, focusing on the conditions set, on the parameters evaluated and on the conclusions of the works.

Author	Aim of the study	Simulation parameter	Parameter evaluated	Conclusion/clinical outcome
Lu *et al.*,^11^ 2016	Prediction of the position of the endoleak formation in post-operative EVAR patients using FSI	One cardiac cycle simulated inlet BC: physiological velocity waveform Outlet BC: physiological pressure waveform	Von Mises stresses distribution. Correlation between endoleak and peak von Mises stresses.	Observation of high correlation between peak stresses and endoleak presence.
Jayendiran *et al.*,^9^ 2020	Estimation of the effect of the flow on the mechanical behaviour of aorta and stent graft, performing an FSI simulation on a honeycomb stent graft.	Continuity condition of displacement and normal stresses applied at the interface. Inlet BC: pressure condition Outlet BC: pressure condition. Implicit technique	Strain during the crimping and release phase. Von Mises stress on the stent graft after expansion using an FSI simulation.	A strain increase as the space between the struts increased, was observed. The sealing stresses on the stent were below the yield stress of the material, and the stent adhered well to the walls.
Bologna *et al.*,^8^ 2023	Development of a patient specific FSI simulation to evaluate flow pattern, pressure, and WSS to understand the benefit of using patient specific stent graft.	FEA BC: load CFD BC: pressure FSI performed using a parallel coupling scheme	CFD: WSS, pressure, velocity, and the flow pattern at peak systole. FEA: wall stress distributions. FSI: pressure, wall stress.	Customised stent grafts for treating AAA satisfied the expected benefits, after both FEA and CFD simulations.
Xie *et al.*,^10^ 2024	Evaluation of the haemodynamic performance of different cross limb features using one way FSI analysis.	One cardiac cycle simulated inlet BC: velocity waveform Outlet BC: pressure waveform FSI is performed using the coupling algorithm.	Flow pattern, helicity strength, DF, and WSS	Increase in the helical strength in the cross limb configuration. An increase in the main body length caused an increase in the pressure drop between inlet and outlet, in the helical flow and vortex. Reducing the cross angle a faster fluid was observed. Increasing the length of the main body and decreasing the cross angle of the limb the displacement forces could be reduced.
Mo *et al.*,^33^ 2025	Evaluation of the blood flow pattern in the aneurysmal sac in the presence of a type II endoleak and the risk of thrombus formation. A comparison between CFD and FSI simulation has been presented to understand differences between the two methods	Three cardiac cycles simulated Inlet BC: pressure waveform Outlet BC: pressure waveforms	Flow velocity, volume flow rate, aneurysm sac pressure, WSS	The higher the volume flow rate and the velocity, the more severe is the endoleak. The flow entered in the aneurysm sac, but it was not unidirectional and depended on the pressure difference between the sac and the branches. CFD and FSI results were in agreement; however, the CFD method overestimated the velocity of the fluid and thus the potential thrombus risk formation.

AAA = abdominal aortic aneurysm; BC = boundary condition; CFD = computational fluid dynamics; DF = displacement forces; EVAR = endovascular aortic repair; FEA = finite element analysis; FSI = fluid–structure interaction; WSS = wall shear stress.

## DISCUSSION

The main goal of this scoping review was to examine the methods identified in the literature for simulating the EVAR procedure *in silico*. This includes CFD simulations to analyse blood behaviour, FEA simulations to investigate the structural behaviour of stent graft and aorta, and FSI analysis to couple the structural and fluid domains.^3^^,^^4^

Most studies used CFD to evaluate post-EVAR haemodynamics, with broadly consistent approaches to boundary conditions and blood modelling.^9^^,^^13^^,^^15^^,^^17^^,^^19^, ^20^, ^21^, ^22^, ^23^, ^24^, ^25^, ^26^, ^27^, ^28^, ^29^, ^30^, ^31^^,^^34^ This methodological consistency makes CFD the most mature technique, and its clinical relevance lies in anticipating flow related complications. Several studies also compared cross limb (‘ballerina’) *vs*. standard configurations, reporting systematic differences in pressure drops and displacement forces, whereas others showed how device specific flow fields affected sac pressurisation and secondary flows.^15^^,^^19^^,^^20^^,^^22^^,^^26^ FEA studies instead aimed to reproduce device deployment and apposition, but showed substantial variation in geometry, material properties, and modelling steps. Despite this heterogeneity, FEA provided clinically relevant insights. Hemmler *et al.*^14^ linked oversizing to increased wall strain at the seal zone, while Perrin *et al.*^18^ validated displacement based deployment against clinical CT data. These results illustrate how FEA can inform sizing and sealing decisions, which can be linked to type Ia or III endoleak and migration risk. Notably, Abdollahi *et al.*^32^ demonstrated a more realistic reproduction of the entire deployment sequence, including crimping, tracking, and release. Such approaches offer a realistic reproduction of the clinical procedure, which can give insight not only into the final apposition of the device but also during the delivery phase, such as the impact of the catheter on the vessel wall. FSI studies were fewer and more heterogeneous, but they uniquely integrated haemodynamics with structural deformation.

Beyond these immediate applications, simulations can contribute to broader aspects of EVAR planning and follow up. Kyriakou *et al.*^13^ linked deployment simulations with subsequent haemodynamic analysis, a combined approach that could be extended to testing relining or fenestrated conversions *in silico*. In terms of patient specific haemodynamics, CFD studies demonstrated differences in displacement forces and wall shear between male and female anatomies, indicating how individual vessel geometry influences risk.^24^ Customised device design may also benefit, with Bologna *et al.*^8^ showing that patient specific FSI simulations of tailored endografts altered stress distributions compared with off the shelf models.

Finally, integration of these tools into long term surveillance is a promising avenue. Domanin *et al.*^25^ showed that displacement measured on dynamic CT angiography correlated with haemodynamic forces in CFD simulations, suggesting that imaging follow up could be combined with simulation outputs to stratify surveillance intensity.

Rather than replacing existing imaging based planning, computational models should be viewed as complementary tools that can transform EVAR planning into a reproducible, quantitative, and patient specific process. This shift holds the promise of not only reducing variability and complication rates but also of optimising device choice, landing zone selection, and procedure strategy to achieve more durable EVAR outcomes.

### Future perspectives

An important next step would be to validate these *in silico* results. Validation efforts should follow structured frameworks such as the Verification and Validation guidelines outlined by the American Society of Mechanical Engineers in 2018.^36^ These Verification and Validation guidelines provide a stepwise approach to assessing model credibility of computational simulations. This approach, which was used by Ramella *et al.*^37^ for thoracic EVAR, demonstrates that high fidelity simulations can be benchmarked against both experimental and clinical data.

Future work should also address practical validation aspects, including reproducibility across centres, sensitivity to input imaging quality, and consistency of outputs when models are applied to different endograft designs or anatomic scenarios.^36^ Finally, integration of simulation validation into prospective registries and clinical trials could accelerate translation, ensuring that *in silico* models evolve from research tools into clinically credible adjuncts for EVAR planning.

### Limitations

Selection and extraction bias may have been introduced, as the focus was primarily on engineering and methodological aspects, with limited clinical data reported. Most of the included work reported technical outcomes without clinical follow up, which restricts assessment of patient level benefit. Engineering specific databases were excluded to prioritise clinically relevant studies; thus PubMed, Scopus, and Web of Science were selected to capture interdisciplinary work.

Another limitation is the general lack of simulation validation according to American Society of Mechanical Engineers standards and the limited clinical validation across studies. Although some models were compared with anatomic images,^18^^,^^32^^,^^38^ few were validated against clinical outcomes such as endoleaks or migration.^39^ There is also no standardised protocol for EVAR simulation setup, leading to methodological variability and limiting reproducibility.^12^, ^13^, ^14^^,^^16^^,^^18^^,^^32^

Despite these limitations, this review offers clinicians an overview of key simulation parameters and methodologies.

## CONCLUSION

This scoping review draws attention to the methodologies used to perform FEA, CFD, and FSI simulations of the EVAR procedure. CFD simulations highlighted a more consistent methodology, whereas FEA and FSI approaches were heterogeneous and remain less standardised.

The scoping review provides an overview of the existing methods and strategies used in EVAR simulations and their potential clinical applications, setting the stage for the development of predictive models for EVAR complications. As computational models continue to evolve, there is potential for these simulations to assist clinicians in pre-operative planning and credible risk assessment, ultimately improving treatment decisions, patient specific care, and long term EVAR outcomes.

## CONFLICTS OF INTEREST

J.A.v.H. is or has been a proctor or consultant for Gore Medical, Terumo Aortic, and Cook Medical. S.T. is a consultant and speaker for Medtronic Inc., W. L. Gore, and Terumo Aortic. To minimise potential bias, the study selection, screening, and data extraction processes were conducted independently by authors without industry affiliations following pre-defined eligibility criteria. No industry partners were involved in the design, analysis, or interpretation of the findings presented.

This research received no specific grant from any funding agency in the public, commercial, or not-for-profit sectors.
