# Supplementary material for: Abdominal Stent Graft Numerical Models to Virtually Simulate Endovascular Aortic Repair: A Scoping Review
Source: EJVES Vasc Forum. 2026 Feb 12;65:131–46. doi: 10.1016/j.ejvsvf.2026.02.001 (PMC13085093; doi:10.1016/j.ejvsvf.2026.02.001)
Supplement: Multimedia component 1 [file mmc1.pdf]

Supplementary Table S1. Overview of the advantages and disadvantages of the different types of computational simulation, their potential clinical use, the data needed to run the simulation and what is needed for the process of Validation of the simulation. Computational time is also included. This is meant as the time to run the simulation itself without the process of segmentation and setting of the simulation.

Method	Advantages	Disadvantages	Clinical Use Potential	Required input	Validation	Computational time
CFD (Computational Fluid Dynamics) [3]	Relatively established; can assess post-EVAR haemodynamics; good for comparing devices	Assumes rigid vessel walls; does not capture structural response; limited for procedural planning	Medium (risk assessment, flow-related complications, endoleak formation, device migration)	Post-CT scan, pressure and velocity data of the patient	Reproduction of the flow condition in rigid, patient-specific phantoms, to validate CFD simulations	5 to 7 hours
FEA (Finite Element Analysis) [3]	Models mechanical behaviour of device and aorta; suitable for deployment simulation	Fluid dynamics is not taken into consideration. Not standardised	High (device sizing, fit prediction, contact forces and pressure assessment)	Pre-and post-CT scan of the patient	Validation with post-operative CT-scan	7 to 10 hours
FSI (Fluid-Structure Interaction) [4]	Captures realistic interaction between flow and structure; most physiologically accurate	Computationally expensive; less widely adopted; model assumptions vary greatly	High (future potential for digital twins, flow related complications, blood-device interaction, aorta and device displacement)	Post-CT scan, pressure and velocity data of the patient	Reproduction of the flow condition in deformable patient-specific phantoms, to validate CFD simulations	1 to 2 days
