# Supplementary material for: Abdominal Stent Graft Numerical Models to Virtually Simulate Endovascular Aortic Repair: A Scoping Review
Source: EJVES Vasc Forum. 2026 Feb 12;65:131–46. doi: 10.1016/j.ejvsvf.2026.02.001 (PMC13085093; doi:10.1016/j.ejvsvf.2026.02.001)
Supplement: Multimedia component 2 [file mmc2.pdf]

Supplementary Table S2. Full search string used per database and concomitant found results

Database	*Search query*	*Results*
1. Pubmed	31 July 2024	280
	(("virtual" OR "simulation*" OR "numerical simulation*" OR "numerical model" OR "finite element method" OR "finite element analysis" OR "computational" OR "computational simulation" OR "computational model")) AND (("EVAR" OR "endovascular aortic repair" OR "abdominal stent-graft" OR '(abdominal stent-graft deployment[tiab])' OR "abdominal stent graft" OR "abdominal endograft" OR "aortic endograft*"))<br>➔ Only the last 10 years	
2. Scopus	31 July 2024	201
	(TITLE-ABS-KEY ("Virtual" OR "simulation*" OR "numerical simulation*" OR "numerical model" OR "finite element method" OR "finite element analysis" OR "computational" OR "computational simulation" OR "computational model")) AND (TITLE-ABS-KEY ("EVAR" OR "abdominal endovascular aortic repair" OR "abdominal stent-graft" OR "abdominal stent-graft deployment" OR "abdominal stent graft" OR "abdominal endograft" OR "aortic endograft*"))<br>➔ Only the last 10 years	
3. Web of Science	31 July 2024	607
	1<br>((((((((ALL=(Virtual)) OR ALL=(simulat*)) OR ALL=(numerical simulation)) OR ALL=(numerical model)) OR ALL=(finite element method)) OR ALL=(finite element analysis)) OR ALL=(computational)) OR ALL=(computational simulation)) OR ALL=(computational model))<br>2<br>((((((ALL=(EVAR)) OR ALL=(Endovascular aortic repair)) OR ALL=(abdominal stent-graft)) OR ALL=(abdominal stent-graft deployment)) OR ALL=(abdominal stent graft)) OR ALL=(abdominal endograft)) OR ALL=(aortic endograft))<br>#2 AND #1<br>➔ Only the last 10 years	
