# Supplementary material for: Abdominal Stent Graft Numerical Models to Virtually Simulate Endovascular Aortic Repair: A Scoping Review
Source: EJVES Vasc Forum. 2026 Feb 12;65:131–46. doi: 10.1016/j.ejvsvf.2026.02.001 (PMC13085093; doi:10.1016/j.ejvsvf.2026.02.001)
Supplement: Multimedia component 3 [file mmc3.pdf]

Supplementary Table S3. Complete quality assessment score per included study

Author, year	Introduction/ background/ rationale	Literature review	Problem statement / objective of the study/ research question	Guiding conceptual or theoretical framework	Study design	Strength of study design: quantitative	Strength of study design: qualitative	Sample and setting	Simulation development	Description of Simulation Implementation	Description of simulation feedback or debriefing	Study instruments: quantitative	Study instruments: qualitative	Results	Discussion	IRB approval /exemption	Quality Assessment Score
Georgakarakos, 2014	1	2	2	1	2	N/A	2	1	1	1	N/A	N/A	1	2	2	N/A	38%
Perrin, 2015	2	2	2	3	3	N/A	4	2	4	3	N/A	N/A	3	4	4	N/A	75%
Lu, 2016	2	2	2	2	2	N/A	2	2	1	1	N/A	N/A	2	1	3	N/A	46%
Stefanov, 2016	1	2	3	3	3	N/A	3	2	2	3	N/A	N/A	4	3	4	N/A	69%
Polanczyk, 2016	3	2	3	3	2	N/A	4	3	2	2	N/A	N/A	3	3	3	N/A	69%
Aristokleous, 2016	3	3	3	3	3	N/A	2	3	3	3	N/A	N/A	4	3	3	N/A	75%
Looyenga, 2017	2	1	2	3	2	N/A	2	1	3	2	N/A	N/A	2	2	2	N/A	50%
Raptis, 2017	1	2	2	1	3	N/A	3	3	1	1	N/A	N/A	2	3	4	N/A	54%
Raptis, 2018	1	1	2	1	3	N/A	3	3	2	2	N/A	N/A	3	3	3	N/A	56%
Liu, 2018	2	2	2	3	3	N/A	3	2	3	3	N/A	N/A	3	3	4	N/A	69%
Hemmler, 2018	3	3	3	4	3	N/A	3	2	4	4	N/A	N/A	4	4	3	N/A	83%
Pocivavsek, 2020	2	2	2	3	3	N/A	3	2	3	3	N/A	N/A	3	3	3	N/A	67%
Jayendiran, 2020	3	3	2	2	3	N/A	3	1	3	3	N/A	N/A	3	4	2	N/A	67%
Kyriakou, 2020	2	2	2	4	4	N/A	4	1	4	3	N/A	N/A	3	4	3	N/A	75%
Pionteck, 2020	2	3	3	4	4	N/A	3	2	4	4	N/A	N/A	3	3	2	N/A	77%
Kyriakou, 2020	2	3	3	4	4	N/A	4	2	4	4	N/A	N/A	3	4	4	N/A	85%
Domanin, 2020	4	3	4	3	3	N/A	3	3	4	3	N/A	N/A	4	4	4	N/A	88%
Qing, 2021	2	3	2	3	3	N/A	3	3	2	2	N/A	N/A	2	3	3	N/A	65%
Ashraf, 2021	2	1	2	2	4	N/A	3	2	4	4	N/A	N/A	3	3	3	N/A	69%
Teng, 2022	2	1	2	3	2	N/A	2	2	2	2	N/A	N/A	2	1	2	N/A	48%
Qing, 2022	2	3	2	3	2	N/A	3	3	2	2	N/A	N/A	3	3	3	N/A	65%

Polanczyk, 2022	3	2	3	4	3	N/A	4	3	3	3	N/A	N/A	4	4	3	N/A	81%
Brand, 2023	2	2	2	2	2	N/A	2	2	2	2	N/A	N/A	3	3	3	N/A	56%
Bologna, 2023	3	3	2	4	3	N/A	3	2	4	4	N/A	N/A	3	2	3	N/A	75%
Xie, 2024	2	2	2	3	2	N/A	3	2	2	2	N/A	N/A	2	3	2	N/A	56%
Zhang, 2024	2	2	1	2	2	N/A	2	1	1	2	N/A	N/A	2	2	2	N/A	48%
Mo, 2025	2	2	3	2	2	N/A	2	3	2	2	N/A	N/A	3	2	2	N/A	56%
Abdollahi, 2025	2	3	3	4	4	N/A	3	3	4	4	N/A	N/A	3	4	4	N/A	85%
