# Supplementary material for: Abdominal Stent Graft Numerical Models to Virtually Simulate Endovascular Aortic Repair: A Scoping Review
Source: EJVES Vasc Forum. 2026 Feb 12;65:131–46. doi: 10.1016/j.ejvsvf.2026.02.001 (PMC13085093; doi:10.1016/j.ejvsvf.2026.02.001)
Supplement: Multimedia component 4 [file mmc4.pdf]

Supplementary Table S4. Clinical data of included studies

Study name	*Publication year*	*Number of patients (N)*	*Disease treated*	*Number of Male patients*	*Age in years +- SD*	*Aneurysm Size in cm +-SD*
**CFD**						
Georgakoros	2014	1	AAA	-	-	-
Aristokleous	2016	4	AAA	4	71 (+-4.1)	-
Polancyzk	2016	27	AAA	-	66.5 (+-6.6)	-
Stefanov	2016	8	AAA	-	-	-
Looyenga	2017	-	-	-	-	-
Raptis	2017	2	AAA	-	-	-
Liu	2018	-	-	-	-	-
Raptis	2018	-	-	-	-	-
Domanin	2020	-	-	-	-	-
Kyriakou	2020	-	-	-	-	-
Ashraf	2021	-	-	-	-	-
Qing	2021	-	-	-	-	-
Polancyzk	2022	-	-	-	-	-
Qing	2022	20	AAA	-	-	-
Teng	2023	-	-	-	-	-
Brand	2023	-	-	-	-	-
Zhang	2024	-	-	-	-	-
**FEA**						
Perrin	2015	3	AAA	3	68.7 (++8.2)	-
Hemmler	2018	-	-	-	-	-
Pocivavsek	2019	1	Ruptured AAA	-	-	-
Kyriakou	2020	-	-	-	-	-
Pionteck	2020	1	AAA	1	78	-
Abdollahi	2025	1	AAA	1	74	5.7
**FSI**						
Lu	2016	6	Endoleak after EVAR	-	81.5 (+-9.0)	6.9 (+-1.4)
Jayendiran	2020	-	-	-	-	-
Bologna	2023	1	AAA	-	-	-
Mo	2025	-	-	-	-	-

AAA = abdominal aortic aneurysm, EVAR = endovascular aortic repair.
