# Supplementary material for: Abdominal Stent Graft Numerical Models to Virtually Simulate Endovascular Aortic Repair: A Scoping Review
Source: EJVES Vasc Forum. 2026 Feb 12;65:131–46. doi: 10.1016/j.ejvsvf.2026.02.001 (PMC13085093; doi:10.1016/j.ejvsvf.2026.02.001)
Supplement: Multimedia component 5 [file mmc5.pdf]

Supplementary Table S5. Methodological characteristics of the Finite Element Analysis (FEA) simulations, focusing on the model discretisation

Author, year	Stent-graft type	Stent-graft Model	Stent-graft Material	Patient-specific - idealised anatomy	Aortic Model	Aortic Material
**Perrin,** 2015 [18]	Medtronic stent-graft and 1 iliac component by Cook	Stent: linear beams<br>Graft: 4-node shell elements	Stent: Auricchio model<br>Graft: orthotropic elastic material	3 patient-specific cases with pre-operative and 1-month post-operative CT.	Linear shell elements.	A linearised model of the Holzapfel-Gasser-Ogden anisotropic hyperelastic model.
**Hemmler,** 2018 [14]	-	Stent: linear hexahedral element<br>Graft: solid hexahedral element	Stent: elastic material<br>Graft: compressible Neo-Hookean model	Idealised model	Linear hexahedral elements	Physiological tract: anisotropic, hyperelastic 2-fiber model (Gasser)<br>Pathological tract: isotropic material (Raghavan and Vorp)
**Kyriakou,** 2020 [13]	Terumo device	Stent: beam enclosed by 4-node quadrilateral surface elements<br>Graft: Kirchhoff thin shell	Stent: linear elastic material, with ring-dependent stiffness<br>Graft: Elastic and Poisson modulus taken from literature	idealised		rigid material
**Pionteck,** 2020 [16]	-	Stent: linear beams<br>Graft: -	Stent: linear elastic material<br>Graft: -	Patient-specific pre and post-CT	-	-
**Pocivavsek,** 2020 [12]	-	Stent: linear tetrahedral elements<br>Graft: linear tetrahedral elements	Stent: elastic Hookean solid model<br>Graft: elastic Hookean solid model	1 patient-specific CT pre and post	Linear tetrahedral elements	Raghavan and Vorp polynomial hyperelastic model.
**Abdollahi,** 2025[32]	Zenith stent-graft (Cook Medical)	Stent: linear elastic<br>Graft: transversely isotropic material	Stent: beam element<br>Graft: quadrilateral element	Pre and post patient-specific CT	3-Node shell element	Second order hyperelastic, homogenous, isotropic and incompressible material
