# Supplementary material for: Abdominal Stent Graft Numerical Models to Virtually Simulate Endovascular Aortic Repair: A Scoping Review
Source: EJVES Vasc Forum. 2026 Feb 12;65:131–46. doi: 10.1016/j.ejvsvf.2026.02.001 (PMC13085093; doi:10.1016/j.ejvsvf.2026.02.001)
Supplement: Multimedia component 6 [file mmc6.pdf]

Supplementary Table S6. Study characteristics of the Fluid-Structure Interaction (FSI) simulation, in particular on the model discretisation, which considers the structural and the fluid part interactions.

Author, year	FSI type	Stent-graft model	Stent-graft material	Input data	Aorta model	Aorta material	Blood model
**Lu,** 2016 [11]	Two-way FSI	Stent: Shell<br>Graft: Shell	Stent/Graft: hyperelastic homogeneous incompressible isotropic material	6 patient-specific CT	Triangular shell elements.	Hyperelastic homogeneous incompressible isotropic material	Incompressible, Newtonian, homogeneous.
**Jayendiran,** 2023[9]	Two-way FSI	Stent: linear triangular shell<br>Graft: linear quadrilateral shell	Graft: neo-Hookean material (anisotropic hyperelastic model).<br>Stent: shape memory material (Lagoundas models)	Idealised anatomy	Linear quadrilateral shell elements	Hyperelastic material using the Holzapfel-Gasser-Odgen model.	Incompressible, Newtonian, homogeneous.
**Bologna,** 2023 [8]	One-way FSI	Stent: tetrahedral<br>Graft: tetrahedral	Graft: linear elastic material<br>Stent: Auricchio and Taylor model	Patient-specific CT	Shell elements	Neo-Hookean model mimicking hyperelasticity	Incompressible, Newtonian
**Xie,** 2023 [10]	One-way FSI			One patient-specific CT-post and 4 idealised models starting from the CT			Incompressible, Newtonian
**Mo,** 2025 [33]	Two-way FSI	- Graft: tetrahedral	-	Idealised model of aorta and graft	- Tetrahedral elements	- Isotropic, linear elastic material	Non- Newtonian fluid (Carreau-Yasuda model)
